# Value of appendicular skeletal muscle mass to total body fat ratio in predicting obesity in elderly people: a 2.2-year longitudinal study

**DOI:** 10.1186/s12877-020-01540-9

**Published:** 2020-04-19

**Authors:** Yu-Jie Zhang, Shi-Hui Fu, Jing-Xin Wang, Xin Zhao, Yao Yao, Xiao-Ying Li

**Affiliations:** 1 Department of Epidemiology, School of Public Health, Southern Medical University, Guangzhou, Guangdong China; 2 Department of Geriatric Cardiology, Chinese PLA General Hospital, Beijing, China; 3 Department of Rehabilitation, Zhengzhou Central Hospital Affiliated to Zhengzhou University, Zhengzhou, China; 4 Department of Rehabilitation, Chinese PLA General Hospital, Beijing, China; 5 International Medical Center, Chinese PLA General Hospital, Beijing, China; 6 Center for Healthy Aging and Development Studies, National School of Development, Peking University, Beijing, China; 7 Institute of Geriatrics, National Clinical Research Center for Geriatric Diseases, Chinese PLA General Hospital, Beijing, China

**Keywords:** Appendicular skeletal muscle mass, Elderly, Obesity, Total body fat

## Abstract

**Background:**

Obesity is a disease characterized by much fat accumulation and abnormal distribution, which was related to cardiovascular diseases, diabetes mellitus (DM) and muscular skeletal diseases. The aim of this study was to evaluate the usefulness of appendicular skeletal muscle mass to total body fat ratio (ASM/TBF) in screening for the risk of obesity in elderly people.

**Methods:**

A prospective study was carried out with 446 participants (non-obese elderly people with body mass index (BMI) < 28 kg/m^2^) who underwent baseline and an average around 2.2-year follow-up health check-up examinations.

**Results:**

The mean age at baseline was 63.6 years. The incidence of new obesity was 5.4% during follow-up. Linear regression demonstrated that baseline ASM/TBFs were negatively correlated with follow-up BMIs in both men and women (*β* = − 1.147 (− 1.463—-0.831) for men and − 4.727 (− 5.761—-3.692) for women). The cut-off points of baseline ASM/TBF in elderly people for obesity were 1.24 in men and 0.90 in women which were identified by Classification and Regression Tree (CART). Logistic regression showed that both men and women with decreased ASM/TBF had higher risks of obesity over the follow-up period (*Relative Risk* (*RRs*) = 5.664 (1.879–17.074) for men and 34.856 (3.930–309.153) for women).

**Conclusions:**

Elderly people with a low ASM/TBF had a higher risk of new obesity, which suggested that ASM/TBF should be considered in obesity management in the elderly.

## Background

Obesity is a disease characterized by much fat accumulation and abnormal distribution, which is related to cardiovascular diseases, diabetes mellitus (DM) and muscular skeletal diseases [1]. Severe obesity has been proved to be a risk factor for shortened life expectancy and poor prognosis [2]. As the population is aging, obesity is becoming more prevalent among elderly people [3]. Despite body mass index (BMI) can somehow reflect integral fatness among individuals, it may make false estimating over adiposity because of fat deposition in viscera or not [4, 5].

The close relationship between appendicular skeletal muscle mass (ASM) / total body fat (TBF) and sarcopenia obesity, which was defined by top TBF quartile, has been well recognized in previous studies [6]. However, the association between this ratio and new obesity has not been adequately investigated. Besides, it is still not known whether ASM/TBF has significant value to access the risk of obesity.

ASM and TBF, represented muscle and fat components and measured by Bioelectric impedance (BIA) technique, were shown after safe current passed through the human body [7, 8]. Previous studies indicated that TBF/weight was a better predictor of cardiovascular risk factors than BMI [7], partly because visceral fat, increasing gradually with aging, was more relevant to TBF. Besides, another study suggested that ASM/TBF was more related to cardio-metabolic and functional abnormalities than TBF/weight, which indicated that ASM/TBF was worth further exploring [9]. However, there was no agreement about the cut-off points for ASM/TBF to diagnose obesity let alone predict it.

Although ASM/TBF has been demonstrated previously [6], little is known about its role in simple obesity assessment. It is necessary to determine whether low ASM/TBF is a risk factor for obesity, which may decrease the quality of life in elderly people. Therefore, the aim of the study was to evaluate the usefulness of ASM/TBF in screening for the risk of obesity in elderly people.

## Methods

### Subjects

Elderly people aged over 60 years who underwent a routine health check-up in Chinese PLA General Hospital from February 2013 to February 2014 were enrolled in this study. The inclusion criteria were elderly people aged over 60 who accepted BIA and anthropometric measurements. Participants were excluded for any of those cases: (a) extremities diseases; (b) previous obesity; (c) diagnosed metastatic advanced tumor or malignant tumor discovery less than 5 years; (d) secondary obesity caused by several diseases, such as Cushing syndrome. Five hundred and forty-five participants were recruited at baseline. Visits were performed at baseline and the next before February 2016. The follow-up time was between 0.6–3.8 years. Finally, a total of 446 participants completed the average around 2.2 years follow-up. The missing rate was 18.2%. All participants were given written informed consents and the study protocol was approved by the Institutional Review Board of Chinese PLA General Hospital.

### Data collection and measurements

Past history (e.g., obesity) and personal history (e.g., smoking habit) were collected by trained healthcare providers. The method of BIA measurement can be seen from our teams’ previous works [10, 11]. Other anthropometric indicators were on standardized tests [12].

### Diagnostic definitions

The definition of smoking history was based on Report on Cardiovascular Diseases in China [13]. Obesity was defined as BMI ≥ 28 kg/m^2^ [14]. The definition of DM was based on Chinese Society of Diabetes (2013 edition) [15].

### Statistical analysis

Data were analyzed by the Statistical Package for Social Sciences, version 22.0 (SPSS, Inc., Chicago, IL, USA). Continuous variables under normal distribution and categorical data were expressed as mean [standard deviation (SD)] and count (percentage), respectively. Classification and Regression Tree (CART) Models, measured in terms of true classification rate of the classifier, were built through a process known as binary recursive partitioning, which is an iterative process that splits the data into partitions or branches, and then continues splitting each partition into smaller groups as the method moves up each branch. The purpose of the analyses via tree-building algorithms was to determine a set of if-then logical (split) conditions that permit accurate prediction or classification of cases. Cut-off values of baseline ASM/TBF for obesity were identified by CART. Moreover, logistic regression was used to examine *Relative Risk* (*RRs*) of new obesity in subjects with low ASM/TBF determined by CART. A two-sided *P* value < 0.05 was considered statistically significant.

## Results

### Participants’ clinical characteristics

The average age at baseline was 63.6 years. The minimum age was 60 and the maximum age was 81. The incidence of new obesity was 5.4% during follow-up. Subjects with new obesity had significantly lower baseline ASM/TBF than subjects without new obesity (*P* < 0.001 in women and *P* = 0.119 in men, Table 1).

**Table 1:** Participants’ clinical characteristics

	Non obesity		New obesity	
	Male (*n* = 250)	Female (*n* = 172)	Male (*n* = 15)	Female (*n* = 9)
Age (years)	63.8 (4.1)	63.5 (3.4)	64.4 (5.2)	62.4 (2.5)
Smoking history (*n*,%)	146 (58.4)	12 (7.0)	9 (60.0)	1 (11.1)
BMI (kg/m^2^)	24.08 (1.94)	23.54 (2.21)	26.46 (0.91)^**^	26.32 (0.74)^††^
Follow-up BMI (kg/m^2^)	24.21 (2.00)	23.67 (2.14)	27.83 (0.41)^**^	28.12 (0.45)^††^
WHR	0.94 (0.04)	0.83 (0.04)	0.97 (0.03)^*^	0.86 (0.01)^††^
Follow-up WHR	0.95 (0.04)	0.84 (0.03)	0.99 (0.05)^**^	0.89 (0.02)^††^
ASM (kg)	25.03 (6.11)	18.45 (2.48)	27.33 (2.46)	18.72 (2.24)
Follow-up ASM (kg)	25.11 (2.66)	18.36 (1.78)	28.07 (2.94)^**^	19.83 (1.99)^†^
TBF (kg)	16.15 (3.77)	17.94 (3.64)	20.59 (3.21)^**^	22.22 (2.55)^†^
Follow-up TBF (kg)	16.53 (3.74)	18.52 (3.44)	22.15 (3.56)^**^	25.27 (2.08)^††^
ASM/TBF (%)	1.67 (0.76)	1.07 (0.27)	1.36 (0.23)	0.84 (0.06)^††^
Follow-up ASM/TBF (%)	1.61 (0.47)	1.02 (0.18)	1.32 (0.37)^*^	0.79 (0.05)^††^
Albumin (g/L)	45.15 (2.59)	45.44 (2.45)	44.71 (3.02)	46.94 (2.03)
Follow-up albumin (g/L)	44.73 (2.45)	44.79 (2.67)	44.08 (2.71)	46.08 (2.51)
DM (*n*, %)	49 (19.6)	38 (22.1)	3 (20.0)	1 (11.1)

Continuous data were shown as mean (SD) and categorical data were *n* (%)

Abbreviations: *BMI* body mass index; *WHR* waist-hip ratio; *AS* appendicular skeletal muscle mass; *TBF* total body fat; *DM* diabetes mellitus

Non obesity versus New obesity: Male: ^*^P < 0.05, ^**^P < 0.001; Female: ^†^*P* < 0.05, ^††^*P* < 0.001

### Effect of baseline ASM/TBF on follow-up BMI

Linear regression demonstrated that baseline ASM/TBFs were negatively correlated with follow-up body mass indexes (BMIs) in both men and women (*β* = − 1.147 for men and − 4.727 for women even after adjusted for age, albumin and DM history; all *P* < 0.001, Table 2).

**Table 2:** Effect of baseline ASM/TBF on follow-up BMI

	Male		Female	
	*β (SE)*	*P* value	*β (SE)*	*P* value
Model 1	−1.150 (0.161)	< 0.001	−4.744 (0.526)	< 0.001
Model 2	−1.141 (0.159)	< 0.001	−4.748 (0.526)	< 0.001
Model 3	−1.147 (0.161)	< 0.001	−4.727 (0.524)	< 0.001

Abbreviations: *SE* standard error; *BMI* body mass index; *ASM* appendicular skeletal muscle mass; *TBF* total body fat

Model 1 was not adjusted for any factor. Model 2 was adjusted for age. Model 3 was adjusted for age, albumin, diabetes history

### Cut-off point of baseline ASM/TBF for future obesity

**Fig. 1:**
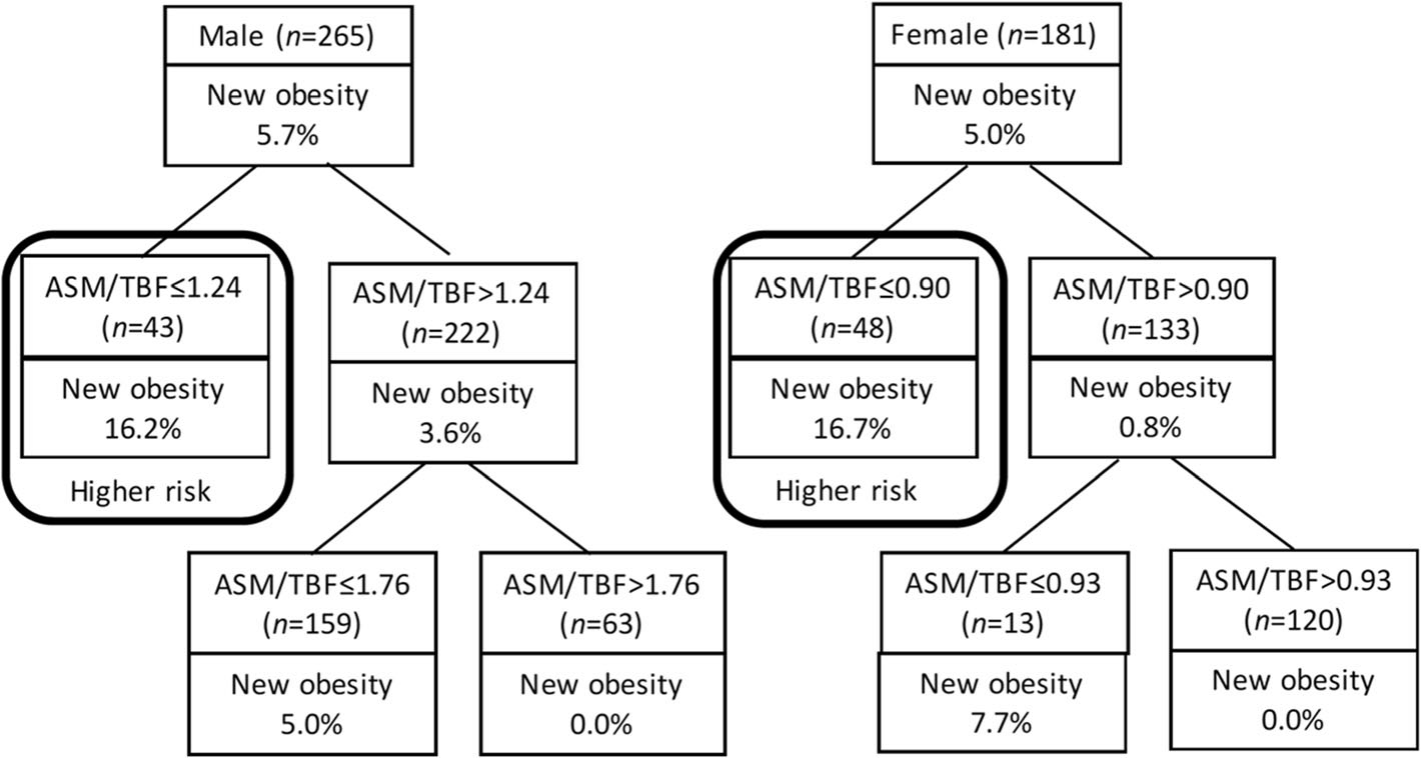
Classification and regression tree models for new obesity on ASM/TBF. Abbreviation: ASM: appendicular skeletal muscle mass; TBF: total body fat

The cut-off points of baseline ASM/TBF in elderly people for obesity were 1.24 in men and 0.90 in women which were identified by CART. Based on cut-off points, decreased ASM/TBFs were defined as less than 1.24 in men and 0.90 in women (Fig. 1).

### Relationship between low baseline ASM/TBF and new obesity

Logistic regression expounded that both men and women with decreased baseline ASM/TBF had higher risk of obesity over follow-up period (*RR* in model 6 were 5.664 for men and 34.856 for women, *95%* confidence interval (*CI*) in model 6 were 1.879–17.074 and 3.930–309.153, separately) (Table 3).

**Table 3:** Relationship between low baseline ASM/TBF and new obesity

	Male		Female	
	*RR (95%CI)*	*P* value	*RR (95%CI)*	*P* value
Model 4	5.201 (1.777–15.226)	0.003	26.400 (3.205–217.479)	0.002
Model 5	5.271 (1.795–15.481)	0.002	30.363 (3.618–254.852)	0.002
Model 6	5.664 (1.879–17.074)	0.002	34.856 (3.930–309.153)	0.001

Abbreviations: *RR* relative risk; *CI* confidence interval; *ASM* appendicular skeletal muscle mass; *TBF* total body fat

Model 4 was not adjusted for any factor. Model 5 was adjusted for age. Model 6 was adjusted for age, albumin, diabetes history

### Relationship between longitudinal low ASM/TBF and new obesity

There were 58 participants (13.0%) with decreased ASM/TBF both at baseline and follow-up. Logistic regression showed that participants with decreased ASM/TBF both at baseline and follow-up demonstrated higher risk of obesity (*RR* in model 9 were 9.299 for men and 69.637 for women, *95% CI* in model 9 were 2.850–30.341 and 7.200–673.552, respectively) (Table 4).

**Table 4:** Relationship between longitudinal low ASM/TBF and new obesity

L-L	Male		Female	
	*RR (95%CI)*	*P* value	*RR (95%CI)*	*P* value
Model 7	8.593 (2.751–26.837)	< 0.001	44.923 (5.391–374.375)	< 0.001
Model 8	8.444 (2.693–26.477)	< 0.001	55.377 (6.399–479.241)	< 0.001
Model 9	9.299 (2.850–30.341)	< 0.001	69.637 (7.200–673.552)	< 0.001

Abbreviations: *RR* relative risk; *CI* confidence interval; *ASM* appendicular skeletal muscle mass; *TBF* total body fat

Model 7 was not adjusted for any factor. Model 8 was adjusted for age. Model 9 was adjusted for age, albumin, diabetes history

## Discussion

This study reported for the first time that the usefulness of ASM/TBF in predicting for the risk of obesity, which was diagnosed by BMI, in elderly people.

The relationship between fat related indicators and obesity has been widely discussed in previous studies [16]. As BMI cannot distinguish fat mass and lean mass clearly and its prediction is prone to be intervened by the different adipose distribution, using BMI as a predictor for body fatness has been controversial [17]. WHR, another anthropometric index, is not universally acknowledged as an accurate indicator to identify individuals at risk for metabolic syndrome [18]. However, ASM/TBF, had its special advantages in considering functional and metabolic impacts of muscle aging and body fat deposition together.

The opinions of obesity were controversial in the elderly. One study involved elderly men in Australia, defined obesity as more than 30% body fat, showed that simple obesity was a protective factor for frailty [19]. But another prospective study demonstrated that obesity increased the mortality of incident cardiovascular diseases [20]. The protective role of obesity may be related to increased ASM, which was paralleled with increasing trends of TBF in the elderly. High ASM, especially robust lower limb strength, contributed to reducing falls [21]. In our study, we used BMI, which has been universally acknowledged as a golden standard, to diagnose obesity. Obesity people had higher ASM than non obesity regardless of gender, which verified the obesity possibly positive role in older people. Moreover, both men and women with decreased baseline ASM/TBF had higher risk of obesity over follow-up period after adjusted for confounding factors, which further elaborated that ASM/TBF had a relationship with obesity. Thus gender imbalance was not an indispensable factor to ASM/TBF in predicting obesity.

Besides, there was no agreement about cut-off points for ASM/TBF to predict obesity. Jean Woo et al. [6] has reported that low ASM/TBFs were defined as less than 0.9 in men and 0.7 in women for 4-year physical limitation. Besides, 0.9 in men and 0.5 in women as cut-off points for 4-year slow walking speed were also shown in her study. However, in our study, CART was also used to determine baseline ASM/TBF cut-off points. The cut-off points of low ASM/TBF were defined as less than 1.24 in men and 0.90 in women, which meaned ASM/TBF can be used as an indicator in predicting obesity.

From the view of histology and embryology, interestingly, skeletal muscle cells shared a common developmental origin with brown adipocytes, which broke down lipids to generate heat, thus reducing obesity [22]. The potential mechanisms of muscle to adipose tissue were chemical pathways. The bioactive substances produced from muscle affected adipose through endocrine or paracrine [23]. Thus, increased muscle mass had a protective function on overweight [24].

Even though it was a longitudinal study, the work had inevitable limitations. Firstly, as a single center study, its biases were unavoidable. For example, because of Berkson bias, a few number of elderly people aged over 80 was recruited in this study. So whether ASM/TBF was an universal and representative assessing indicator to older people needs further exploration. Secondly, visceral fat mass, which was a potential confounding variable, was not demonstrated in this study, therefore a large and longitudinal cohort is warranted to further demonstrate the relationship between visceral fat mass related indicators and obesity. Thirdly, because the new obese population is so small, the results from logistic regression may not be very reliable, which needs amplified sample size to further verify the predictive value of ASM/TBF in obesity.

## Conclusion

Elderly people with decreased ASM/TBF had higher risk of new obesity, which suggested that ASM/TBF should be considered in obesity management in elderly people.

## Data Availability

In attempt to preserve the privacy of patients, clinical data of patients will not be shared, but will be made publicly available upon request. If possible, ZYJ could be contacted once someone wants to request the data.
